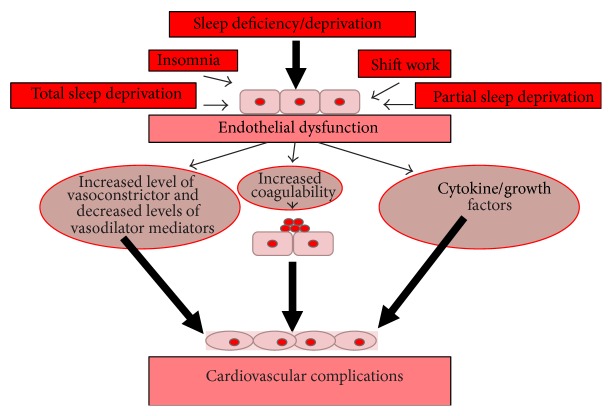# Sleep Deficiency and Deprivation Leading to Cardiovascular Disease

**DOI:** 10.1155/2015/615681

**Published:** 2015-10-01

**Authors:** Michelle Kohansieh, Amgad N. Makaryus

**Affiliations:** ^1^Stern College for Women, Yeshiva University, New York, NY 10016, USA; ^2^Department of Cardiology, North Shore-LIJ Health System, Hofstra North Shore-LIJ School of Medicine, Nassau University Medical Center, East Meadow, NY 11554, USA

## Abstract

Sleep plays a vital role in an individual's mental, emotional, and physiological well-being. Not only does sleep deficiency lead to neurological and psychological disorders, but also the literature has explored the adverse effects of sleep deficiency on the cardiovascular system. Decreased quantity and quality of sleep have been linked to cardiovascular disease (CVD) risk factors, such as hypertension, obesity, diabetes, and dyslipidemia. We explore the literature correlating primary sleep deficiency and deprivation as a cause for cardiovascular disease and cite endothelial dysfunction as a common underlying mechanism.

## 1. Introduction

Sleep is an essential part of human health and well-being. Sleep plays a vital role in an individual's mental, emotional, and physiological health. Not only does sleep deficiency lead to neurological and psychological disorders, but also vast amounts of literature have explored the adverse effects of sleep deficiency on the cardiovascular system. Decreased quantity and quality of sleep, whether due to sleep disorders or just through lack of proper sleep patterns, have been linked to cardiovascular disease (CVD) risk factors, such as hypertension, obesity, diabetes, and dyslipidemia [1–3]. Studies have shown that short durations of sleep are associated with greater risk of developing or dying from CVD [4]. While secondary causes of sleep deficiency leading to CVD have been well described such as obstructive sleep apnea, here we explore the literature correlating primary sleep deficiency and deprivation as a cause for cardiovascular disease through an underlying mechanism of endothelial dysfunction.

## 2. Sleep Deficiency and Deprivation: Defining the Problem

Sleep deprivation and deficiency have a high prevalence in western societies. The National Sleep Foundation reported that less than half (44%) of all Americans receive a good night's sleep almost every night [5]. According to the National Institute of Health, sleep deficiency is a broad concept that occurs (a) if an individual does not get enough sleep (sleep deprivation), (b) if an individual's sleeping habits are out of sync with the body's natural circadian rhythm (sleeping during the wrong time of the day), and (c) if the quality or quantity of sleep is diminished due to a sleep disorder or external factors [6]. Our review will focus on four specific variations of sleep deficiency: insomnia, acute total sleep deprivation (TSD), partial sleep deprivation (PSD), and night shift workers.

Acute TSD refers to the avoidance of sleep for a period of at least one night. PSD, or sleep restriction, refers to the reduction in the total sleep time relative to one's usual baseline during a 24-hour period. PSD is the most common form of sleep deprivation encountered in everyday life in modern societies [7]. Insomnia is defined as a predominant complaint of dissatisfaction with sleep quantity or quality, associated with one or more of the following symptoms: difficulty initiating sleep, difficulty maintaining sleep characterized by frequent awakenings or problems returning to sleep after awakenings, or early morning awakenings with inability to return to sleep [8]. A shift worker is anyone who follows a work schedule that is outside the typical “9 to 5” business day. According to the Bureau of Labor Statistics, millions of Americans are considered shift workers, including doctors and nurses, pilots, bridge builders, police officers, customer service representatives, and commercial drivers. Such workers often do not sleep in sync with the circadian rhythm, are sleep deprived, and experience frequent sleep disturbances [9, 10].

## 3. Establishing the Link between Sleep Deficiency/Deprivation and Cardiovascular Disease

### 3.1. Endothelial Dysfunction

The endothelium is the thin layer of cells that covers the internal surface of blood vessels, cardiac valves, and several body cavities. These cells play a vital role in maintaining homeostasis by sensing changes in hemodynamic forces and blood-borne signals. In response to homeostatic changes, endothelial cells elicit relaxation and contractions of the underlying vascular smooth muscle cells releasing vasoactive substances. Among those substances, nitric oxide (NO) plays a key role [11].

When an imbalance of the actions of the endothelium toward reduced vasodilation and increased vasoconstriction as well as increased prothrombotic properties occurs, it is said that endothelial dysfunction is present. Arterial endothelial dysfunction is an important event central to the pathogenesis of atherosclerosis. Continued endothelial dysfunction contributes to plaque initiation and progression [12].

Endothelial function can be measured in coronary arteries and in the periphery by measuring vasomotor function after intra-arterial infusion of pharmacologic substances that enhance the release of endothelial NO. The disadvantage of these methods is their invasive nature, which generally makes them unsuitable for studies involving asymptomatic subjects. For this reason, noninvasive tests of endothelial function have been developed and are more commonly used. Flow mediated dilation (FMD) is an ultrasound-based method that measures arterial diameter in response to an increase in shear stress, which causes endothelium-dependent dilatation [13]. This method can be applied more widely for the evaluation of endothelial dysfunction and has been applied to patients with sleep disorders.

### 3.2. Insomnia

One major study, the HUNT 3 (Nord-Trøndelag Health Study) fitness study, has explored the connection between insomnia and endothelial function. The study produced negative results, providing no association between endothelial dysfunction and insomnia. There were no consistent associations between the cumulative number of insomnia symptoms and FMD. However, when the study analyzed individual insomnia symptoms, it found that certain symptoms might be related to endothelial dysfunction and, interestingly, those symptoms differed by gender. Among women, there was an inverse association of early awakenings with endothelial function, but there was an opposite association for men. In addition, women who reported daytime sleepiness had a higher FMD than other women [14].

The HUNT 3 study had followed earlier health studies in Norway including the HUNT study which researched the association between insomnia and ill health and showed that insomnia is a significant risk factor for myocardial infarction [15]. The negative results of the HUNT 3 fitness study were not expected. It should be noted that the study had several limitations that may have led to such results, such as a self-selection bias and the fact that the study restricted itself to individuals free of CVD and hypertension. This introduces a stratification bias excluding a significant population who may exhibit endothelial dysfunction.

### 3.3. Total Sleep Deprivation

In contrast to insomnia, there is more literature on the effects of TSD on endothelial function. One particular study which examined cardiologists on call for 24 hours showed that, after being on call, along with an increase in blood pressure (BP), thirteen out of the fifteen physicians had a brachial artery dilatation that did not reach 4.4%, and five of them did not have any dilation at all [16]. This analysis attributes the difference in endothelial function to stress since it is traditionally accepted that mental stress is linked to activation of the sympathetic nervous system. In this case apparently there was a double stress: stress induced by a lack of sleep and stress secondary to high level medical decision making. The differentiation between the results that were caused due to a lack of sleep and those due to the mental stress of being on call for a long period of time is not clear however. Ghiadoni et al. conducted a study investigating the link between mental stress and endothelial function and found that brief episodes of mental stress, like those encountered in everyday life, may cause transient (up to a period of 4 hours) endothelial dysfunction in healthy young individuals [17].

Another study by Sauvet et al., exploring the effect of acute sleep deprivation on vascular function in twelve healthy males, found that the endothelium-dependent and the endothelium-independent cutaneous vascular reactivity indices were significantly decreased after 29 hours of TSD. By contrast, heart rate, systolic blood pressure, and the normalized low-frequency component of heart rate variability (0.04–0.15 Hz), a marker of sympathetic activity, increased significantly within 32 hours of TSD [18]. This same group of researchers then conducted a follow-up study in rats. They found that TSD induced a reduction in endothelial-dependent vasodilation [19].

### 3.4. Partial Sleep Deprivation

The relationship between PSD and endothelial dysfunction has received more attention than TSD and insomnia. In the several studies performed in the literature, PSD has consistently been linked to decreased vasodilation. Covassin et al. conducted a study on 16 healthy subjects who underwent a 15-day inpatient protocol consisting of a three-day acclimation period, eight days of either sleep deprivation or normal sleep, and four days of recovery. Compared to the acclimation phase during which normal sleep occurred, FMD decreased during the experimental phase in the sleep deprived group (8.6 ± 4.6% versus 5.2 ± 3.4%, *P* = 0.008), while it remained unchanged in controls (5.04 ± 3% versus 6.73 ± 2.94%, *P* = 0.109) [20]. A study conducted by Pugh et al. demonstrated that, compared to the control group who received three nights of full sleep and did not exhibit any changes in their endothelial function, the participants who received three nights of PSD (4 hours of sleep) had a decreased endothelial function by 46.7 ± 1.6% after the second night of sleep restriction but, interestingly, recovered after the third night of PSD [21]. Dettoni et al. observed the effects of PSD for five nights in 13 healthy males. They found a reduction in the maximum endothelial-dependent venodilation (100 ± 22 versus 41 ± 20%) [22].

### 3.5. Shift Work

Compared to the other sleep habits that were mentioned, shift work has received the most attention when considering its effects on endothelial function. One observational study conducted on 22 healthy female nurses showed that after they worked 3 sequential night shifts the FMD was significantly decreased from baseline FMD taken after one regular workday [23]. Suessenbacher et al. compared 48 male shift workers with 47 male nonshift workers from a glass manufacturer using the EndoPAT technique to determine peripheral arterial tone (PAT). They found that, despite a greater percentage of regular physical activity among the shift workers (16.7 versus 4.3%), shift work was associated with a reduced PAT index compared to working only on the day shift (PAT index 1.73 ± 0.4 versus 1.94 ± 0.5) [24]. While physical activity has been associated with better endothelial function [25], this study suggested that the effects of sleep deprivation override the benefits of physical activity on vascular health. Wehrens et al. studied the long term effects of shift work. Their study compared the difference in FMD after two groups (shift workers compared to nonshift workers) were put through sleep deprivation and recovery sleep in identical laboratory settings. After correcting for the difference in body mass index (BMI), there was a trend for lower %FMD (*P* = 0.08) observed among shift workers compared to nonshift workers [26]. Amir et al. conducted a study that had results consistent with this trend. Thirty healthy physicians who had worked night shifts for an average of 5 ± 3 years had their endothelial function examined after a regular workday as the baseline and after a continuous workday of 24 hours including a night shift. Overall, there was a significant decrease in FMD after shift work compared with baseline measurements (6.7 ± 4.8% versus 10.5 ± 4.5%). The authors more importantly also noted that FMD decreased significantly in all subsets except in physicians with a shorter (<3 years) history of night shifts. In these physicians with the shorter history, the change in FMD after the shift was independently related to the length of shift work history [27]. These results were consistent with those found in the previous study by Wehrens et al. Both articles suggested that there may be long term implications of shift work on vascular function.

## 4. Mechanisms of Endothelial Dysfunction Caused by Sleep Deprivation (Figure 1)

### 4.1. Sympathetic Activation

Sympathetic overactivity has been a proposed explanation to the link that is seen between sleep deprivation and endothelial dysfunction. Dettoni et al. attribute the decrease in the maximum endothelial-dependent venodilation found in healthy males after PSD to an increase in sympathetic activity as the participants also experienced an increase in percent low-frequency (50 ± 15 versus 59 ± 8) and a decrease in percent high-frequency (50 ± 10 versus 41 ± 8) components of heart rate variability, increase in low-frequency band of blood pressure variability, and an increase in their serum norepinephrine (119 ± 46 versus 162 ± 58 ng/mL) [22].

Other studies, however, have rejected the association of sleep deprivation and sympathetic activation. Of the nine studies that link sleep deficiency and endothelial function mentioned in our review, three studies had a significant change in blood pressure, four studies did not investigate blood pressure, and two studies saw no difference in blood pressure. Studies that did not show a change in blood pressure, or did show a change but the change came after evidence of endothelial dysfunction, argue that endothelial dysfunction may not be due to increased sympathetic activity of being awake for a prolonged period of time but rather due to another factor. In a study on rats by Sauvet et al., they concluded that while sleep deprivation did decrease endothelial vasodilation, it was not due to changes in blood pressure and was independent of sympathetic activity because it was still evident after pharmacological sympathectomy. Rather, it appears to be associated with NO synthase and cyclooxygenase pathway alterations, specifically, a decrease in the activity of those pathways [19]. The authors, however, mentioned that a persistent increase in sympathetic activity could lead to endothelial dysfunction. This was supported by studies that have shown that subjects with a greater history of night shift work are more likely to have more endothelial dysfunction than subjects who rarely took the night shift and therefore argue the direct causal effect of sympathetic activation [26, 27].

### 4.2. The Role of Nitric Oxide

Endothelial dysfunction is known to be related to the bioavailability of NO which can lead to disruption of vascular homeostasis. NO is responsible for the modulation of vascular dilator tone, regulation of local cell growth, and protection of blood vessels from injurious consequences of platelets and cells circulating in blood. NO therefore plays a crucial role in normal endothelial function [28]. In the study conducted by Suessenbacher et al. on female nurses, in addition to the fact that after working sequential night shifts endothelial function was impaired, the results also showed that mono-nitrogen oxides (NO_*x*_) were also significantly decreased after 3 sequential night shifts compared with the baseline measurements (from 176.1 ± 65.1 mmol/dL to 131.8 ± 72.1 mmol/dL, *P* = 0.033), although, in the end, there was no correlation between changes in NO_*x*_ and FMD before and after 3 sequential night shifts (*r* = −0.218, *P* = 0.356) [24]. In rats, TSD was found to lead to a decrease in NO [29].

It is possible that the reduction in the bioavailability of NO in these sleep deprivation cases may be due to the decreased expression of NO synthase (eNOS) by endothelial cells or a lack of substrate or cofactors for eNOS activity [30, 31]. Altered signaling is also a possibility. However, when considering sleep deficiency, oxidative damage seems to be the mechanism. Oxidative stress occurs when there is an imbalance between oxidizing free radicals and antioxidant defenses. Free radicals or reactive oxygen species (ROS) such as O_2_^−^ are quick to react with and inactivate NO. Thus, vascular oxidative stress can lead to a decrease in NO bioavailability. Under normal physiological conditions, endogenous antioxidant defenses minimize this interaction, thus allowing the body to maintain its ideal amount of NO. Sleep deprivation has been linked to increased uncompensated oxidative stress in peripheral tissues; however, a positive finding shows that recovery sleep can actually restore antioxidant activities [32]. A more recent study found that sleep deprivation does affect antioxidant activity by producing and imbalance in the oxidizing of the spleen cells. While the mechanisms of the cytoxic-like effects of sleep deprivation are likely “related to dysfunction in mitochondrial metabolism and vulnerability in cell signaling pathways,” the exact mechanisms are not understood and require further study [33].

## 5. Conclusion

While there is evidence of an association between endothelial dysfunction and sleep deprivation, it still remains to be evaluated if sleep deprivation is a cause of or is associated with increased risk of CVD. However, endothelial dysfunction is an established independent risk factor for cardiovascular disease. Therefore, many of the factors that link endothelial dysfunction to cardiovascular disease are likely a result of the negative effects of sleep deficiency and deprivation. Further research in the area of sleep deprivation/deficiency is needed especially its relation to cardiovascular disease.

## Figures and Tables

**Figure 1:**